# Feeding Patterns and Postpartum Depressive Symptoms: The Mediating Role of Parenting Self-Efficacy

**DOI:** 10.1155/da/2748707

**Published:** 2025-03-06

**Authors:** Yi Zhu, Yuhang Xie, Xiaoxu Yin, Yanhong Gong

**Affiliations:** ^1^Center for Reproductive Medicine, Department of Gynecology, Zhejiang Provincial People's Hospital (Affiliated People's Hospital), Hangzhou Medical College, Hangzhou, Zhejiang, China; ^2^Department of Institutional Reform and Primary Health, Health Bureau of Shenzhen Guangming District, Shenzhen, Guangdong, China; ^3^Department of Social Medicine and Health Management, School of Public Health, Tongji Medical College, Huazhong University of Science and Technology, Wuhan, Hubei, China

**Keywords:** breastfeeding, mediating role, parenting self-efficacy, postpartum depressive symptoms

## Abstract

**Objective:** The mechanisms underlying the association between breastfeeding and postpartum depressive symptoms remain unclear. In this study, we analyzed the relationship between maternal feeding patterns and postpartum depressive symptoms and investigated the mediating role of parenting self-efficacy (PSE).

**Methods:** A prospective longitudinal survey of 708 mothers was conducted from September 2018 to August 2020. Structural equation modeling was used to examine the relationship between feeding patterns and postpartum depressive symptoms and the effect of PSE.

**Results:** Breastfeeding mothers experienced milder depressive symptoms and higher PSE than women who did not breastfeed. The structural equation modeling results showed a direct effect of feeding patterns on postpartum depressive symptoms and an indirect effect of postpartum depressive symptoms through PSE.

**Conclusions:** Feeding patterns affected postpartum depression through PSE, which enhanced the favorable effects of breastfeeding in preventing postpartum depression and protecting maternal mental health. Future measures aimed at optimizing PSE will not only help prevent postpartum depression but also contribute to further promoting the psychological health and resilience of breastfeeding women.

## 1. Introduction

The risk of depression for women is approximately 20% higher during the postpartum period than during other periods because they are faced with the arduous tasks of infant care and new social roles and expectations [1]. Postpartum depression is a common maternal mental disorder that typically manifests as emotional problems such as anxiety, depressed mood, feelings of worthlessness or guilt, and physical symptoms such as sleep disorders, weight loss, and energy loss [2, 3]. A recent meta-analysis of postpartum depressive symptoms determined that 14.8% of Chinese mothers experienced depressive symptoms after giving birth [4]. Postpartum depression negatively affects the physical and mental health of mothers [5] and is related to negative cognitive, emotional, and behavioral development in infants [6]. Therefore, preventing and managing postpartum depression and improving maternal mental health are important public health priorities.

Parenting self-efficacy (PSE) refers to mothers' efficacy and ability to meet their parenting requirements. PSE is an independent protective factor for postpartum depression [7, 8]. PSE is related to positive maternal–infant interactions and infant attachment, reflecting women's beliefs about effectively performing maternal roles [9]. Women with higher levels of PSE are assured in their parenting skills and feel more at ease when engaging with their babies [10]. In contrast, mothers with lower levels of PSE lack confidence in completing parenting tasks and struggle to achieve satisfaction and comfort in their parenting roles. Research suggested that improving maternal behavioral opportunities and mother-infant intimacy can help enhance their PSE [11], among which breastfeeding may be one of the cost-effective practices.

Breastfeeding has been proven to be an optimal feeding practice and is essential to infant and maternal health. The benefits to children include a reduced risk of infectious diseases, leukemia, and chronic diseases such as diabetes, overweight, and obesity [12]. Breastfeeding women are at a reduced risk of getting diabetes and breast and cervical cancers [13]. In addition to its health advantages, breastfeeding offers economic benefits. According to a 2016 report, if the rate of exclusive breastfeeding during the first 6 months after childbirth increased by 10% in China, the cost of treating illnesses in Chinese children would be reduced by 30 million USD [14]. The benefits of breastfeeding have reached a global consensus, and its protective effects on maternal role adaptation and mental health have begun to attract attention. Notably, early breastfeeding helped mothers adapt to their parenting roles as soon as possible by increasing physical communication and connection with their infants, which helped to improve mothers' confidence and positive emotions in parenting [15].

Strong relationships exist between feeding patterns, PSE, and postpartum depression. Researchers have conducted a series of studies to explore the relationship between these three variables to improve maternal and infant well-being during the postpartum period. To date, however, the results of these studies have been inconsistent. Scholars such as Xia et al. [16] and Leong et al. [17] have suggested that breastfeeding and PSE were protective factors against postpartum depression. Breastfeeding and parenting confidence have also been found to be related to positive maternal-infant interactions and infant attachment. A good maternal role helped alleviate personal pain and negative emotions during parenting [18]. In contrast, a Polish cross-sectional study of infant feeding practices among 151 participants noted that breastfeeding itself may not be the risk factor of postpartum depression. Role processes and perceived role identity experienced by mothers in relation to various feeding styles were critical factors in preventing postpartum depression [19]. Simultaneously, a prospective study in Norway found no direct correlation between feeding patterns and postpartum depression and suggested that some pathways may mediate this relationship [20]. Similar findings have also been reported in the Maldives [21] and Canada [22]. Owing to the variations in socioeconomic and cultural systems in different regions and the cross-sectional design limitations of some studies, it remains unclear whether feeding patterns are directly related to postpartum depression and whether PSE mediates the relationship between these two variables. To explore the interrelationships and influencing paths between these three variables above, it is necessary to conduct relevant large-sample prospective longitudinal studies.

Based on earlier investigations on the relationship between feeding patterns, PSE, and depression, we proposed the following four hypotheses. Feeding patterns are negatively related to PSE, indicating that breastfeeding mothers have better PSE than those who do not breastfeed (Hypothesis 1). PSE is negatively related to postpartum depressive symptoms, indicating that the higher the PSE, the lower the depression level (Hypothesis 2). Feeding patterns not only directly affect postpartum depressive symptoms (Hypothesis 3) but also affect postpartum depressive symptoms through PSE (Hypothesis 4). The aim of this study was to determine the current status of postpartum depressive symptoms and investigate the longitudinal correlation between feeding patterns, PSE, and postpartum depressive symptoms using structural equation modeling. The findings of this study will provide important references for developing strategies to prevent and manage postpartum depression.

## 2. Materials and Methods

### 2.1. Ethics Statement

The Research Ethics Committee of Tongji Medical College at Huazhong University of Science and Technology approved this study (Number: 2016IEC(S084)) and conducted in accordance with the Declaration of Helsinki. Each participant reviewed the investigation's purpose statement and submitted written informed consent.

### 2.2. Study Design and Data Collection

Convenience sampling was used to select a hospital in each city of Hubei Province, China, as the research sites for the survey. The Wuhan and Qianjiang Women's and Children's Hospitals were the two selected. The recruitment and follow-up of the study participants were carried out from September 2018 to August 2020.

The following were the criteria for inclusion: (1) age 18 years or older, (2) full-term birth, (3) married, (4) voluntary participation in the study, and (5) no family history of mental illnesses. Childbirth at full term allows a favorable environment to be created, promoting maternal and infant well-being and attachment. These women are more energetic and capable of engaging in and completing surveys that require multiple measurements [23]. They are more likely to follow medical advice as they have benefited from the process. This, in turn, facilitates subsequent early breastfeeding guidance and postpartum depression intervention. This is why a full-term delivery was one of the inclusion criteria for this study.

A prospective longitudinal research design for data collection was employed based on the existing theoretical framework and research objectives. Data on feeding patterns was collected at 1 month postpartum, whereas PSE and postpartum depression were collected from the study participants at 3 and 6 months postpartum, respectively, to investigate the longitudinal associations among these three factors. Maternal PSE stabilizes around 3 months postpartum when parenting beliefs and satisfaction can be accurately measured [24]. Postpartum depression symptoms can last up to 1 year and are most severe during the first 6 months [2, 6]. Therefore, three longitudinal time points were selected.

A total of 794 mothers participated in the 1-month postpartum survey, of whom 708 completed the 3- and 6-month postpartum follow-ups. The attrition rate was 10.83%, and participants who completed the study and those lost to follow-up did not differ significantly in terms of demographic characteristics, suggesting that our study provided representative results. The reasons for not completing the follow-up survey included being busy with work, childcare tasks, and moving residences. The final sample comprised 708 women.

### 2.3. Procedure

During the recruitment period, women visiting the hospital for standard obstetric exams were invited by the investigators to participate through a face-to-face electronic survey. The survey was conducted via a Chinese online platform called Questionnaire Star (https://www.wjx.cn). It was shared on WeChat, China's most popular social media network, during the follow-up period.

### 2.4. Measures

The questionnaire was designed and distributed to each participant based on a review of the published literature. The questionnaires included questions regarding socio-demographic characteristics, feeding patterns, PSE, and postpartum depressive symptoms. At 1 month postpartum, we collected the socio-demographic characteristics and feeding patterns of the study participants and assessed their PSE and postpartum depressive symptoms at 3 and 6 months postpartum, respectively.

#### 2.4.1. Demographic Information

Demographic information included maternal age, education level (ordinal variable, coded 1 = junior high school and below, 2 = high school, 3 = junior college, 4 = master's degree or higher), work status (0 = employed, 1 = unemployed), and residence (0 = urban, 1 = rural). The confounding effects of demographic factors on the relationship between breastfeeding and postpartum depressive symptoms were controlled. These variables were picked because earlier studies found them to be related to feeding patterns or postpartum depressive symptoms.

#### 2.4.2. Breastfeeding Behaviors

The question “How do you feed the infant?” was used to identify breastfeeding behaviors, with the options “breastfeeding” and “non-breastfeeding” available. Breastfeeding was described as feeding the infant breast milk only or feeding the infant breast milk and formula. Non-breastfeeding implied that the infant was given formula exclusively. Responses were coded as 0 for breastfeeding and 1 for non-breastfeeding.

#### 2.4.3. PSE

The Parenting Sense of Competence Scale was used to measure maternal PSE. This scale was developed by Gibaud-Wallston and Wandersman [25] and was designed to assess parenting self-esteem. The scale is composed of 17 items divided into two subscales: the “Efficacy” subscale, which consists of 8 items to evaluate the perceived competence in parenting knowledge and skills, and the “Satisfaction” subscale, which includes nine items to gauge satisfaction and ease in parenting. Every item is evaluated on a 6-point scale from 1 to 6, with overall scores ranging from 17 to 102 points, 8–48 points for the Efficacy subscale, and 9–54 points for the Satisfaction subscale. The higher the score, the higher the PSE. The Chinese version of this scale has good internal consistency [9]. The scale's internal consistency (Cronbach's *α*) was 0.89 in this study.

#### 2.4.4. Depressive Symptoms

Maternal depressive symptoms were evaluated using the Edinburgh Postnatal Depression Scale (EPDS). The EPDS is a self-report questionnaire consisting of 10 items, created by Cox, Holden, and Sagovsky [26]. This scale covers three dimensions: anhedonia, anxiety, and depression. Each EPDS item is rated from 0 to 3. The highest possible score is 30 points, where higher scores reflect more severe symptoms of depression. We used an EPDS score of ≥13 as the positive screening criterion for PPDs [27]. Cronbach's *α* value of the EPDS Chinese version is 0.78 [28]. The EPDS's internal consistency (Cronbach's *α*) was 0.89.

### 2.5. Statistical Analyses

The Statistical Analysis System (SAS) 9.4 for Windows, developed by SAS Institute Inc. in Cary, NC, USA, was used to conduct statistical analyses. First, the reliability and validity of the survey results were tested. Second, a descriptive analysis was conducted for the participant's basic demographic characteristics using indicators such as mean, standard deviation (SD), and rate or constituent ratio. In addition, *t*-tests or one-way analysis of variance were used to assess differences in depressive symptoms and PSE based on feeding patterns.

The longitudinal structural equation model (SEM) was built using IBM SPSS Amos 26.0 (IBM Corp., Armonk, NY, USA), which was modified according to the fitting index. Structural equation modeling is a causal modeling method based on statistical analysis techniques. Moreover, this modeling can estimate and test abstract concepts that are difficult to measure directly, as well as estimate latent variables and predictive models. Modeling was performed using the asymptotically distribution-free estimation method. The bias-corrected Bootstrap method was used for the mediation effect test, with 5000 resampled samples selected from the original data. Based on this path, the coefficients were tested for significance. If the Bootstrap 95% confidence interval (CI) did not encompass 0, the mediation effect was regarded as significant [29].

Covariate variables such as age, education level, work status, residence, and feeding patterns at 3 and 6 months postpartum were included in the model as control variables. The model's overall fit was assessed using the following fit indices: root mean square error of approximation (RMSEA), incremental fit index (IFI), comparative fit index (CFI), goodness-of-fit index (GFI), adjusted GFI (AGFI), proportion of GFI (PGFI), and parsimony normed fit index (PNFI). RMSEA (95% CI) value < 0.08, CFI, GFI, and AGFI values > 0.90, and PGFI and PNFI values > 0.50 indicated a good fit [30]. All comparisons used a two-tailed approach, with a significance level set at *p* < 0.05.

## 3. Results

### 3.1. Participant Demographic Characteristics

The participants' demographic information is shown in Table 1. The average age of the 708 mothers was 27.14 ± 3.17 years. Most women had junior college diplomas or higher (77.26%), were employed (60.31%), and lived in urban areas (83.76%). The percentage of mothers who breastfed was 51.41%.

### 3.2. Comparison of Postpartum Depressive Symptoms and PSE Characteristics Among Women With Different Feeding Patterns

Table 2 lists the average scores of postpartum depressive symptoms and PSE for different feeding patterns. The mean scores of postpartum depressive symptoms and PSE were 9.72 ± 5.43 and 70.16 ± 12.34, respectively. Based on the criterion, depressive symptoms were identified using a cutoff score of 13. The prevalence of depressive symptoms was 30.56%. The univariate analysis revealed a significant difference in postpartum depression scores among the different feeding patterns. Compared with breastfeeding mothers, women who did not breastfeed had higher levels of depressive symptoms. PSE was also significantly related to feeding patterns. Women who breastfed reported higher PSE than those who did not.

### 3.3. Path Analysis Between Feeding Patterns, PSE, and Postpartum Depressive Symptoms

The SEM was constructed using feeding patterns as the independent variable, postpartum depressive symptoms as the dependent variable, and PSE as the mediating variable. The fitting results of the final model are presented in Table 3. The overall model fit indices of the hypothetical model were RMSEA (95% CI) = 0.066 (0.055, 0.078), IFI = 0.980, CFI = 0.984, GFI = 0.967, AGFI = 0.944, PGFI = 0.599, and PNFI = 0.501, all of which approached the recommended values, indicating that the model fits well.

**Figure 1:**
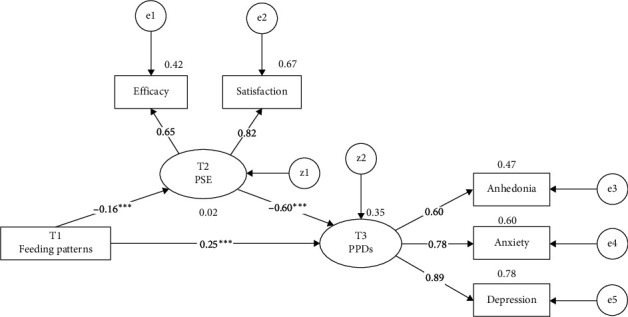
Path diagram of SEM. *Notes:* Coefficients indicate standardized values. Latent variables are illustrated with ovals and manifest variables with rectangles. *⁣*^*∗∗∗*^*p*  < 0.001. PSE, parenting self-efficacy; PPDs, postpartum depression symptoms; SEM, structural equation model; T1, the initial evaluation conducted 1 month postpartum; T2, the second evaluation 3 months postpartum; T3, the third evaluation 6 months postpartum.

The significance of the effect relationship between variables was verified by testing each effect using a bias-corrected Bootstrap. The path coefficients estimated are presented in Figure 1 and Table 4. Feeding patterns were negatively related to PSE (*β* = −0.16), which supported Hypothesis 1. Subsequently, PSE negatively affected postpartum depressive symptoms (*β* = −0.60), which supported Hypothesis 2. The effect (*β* = 0.35) of feeding patterns on postpartum depressive symptoms comprised its direct effect (*β* = 0.25) and its indirect effect (*β* = 0.10 = −0.16 × (−0.60)), mediated through PSE. Therefore, Hypotheses 3 and 4 were supported.

The mediating effect of PSE on the model pathway was validated. Test results of the mediation effect are shown in Table 5. Bootstrapping revealed that the CI estimates of the mediation effect path coefficients did not contain 0, indicating the existence of a mediation effect. Additionally, the mediation effect with a percentage of mediation was 28.57%.

## 4. Discussion

In this study, we aimed to understand the level of postpartum depressive symptoms and investigate the relationship between feeding patterns and postpartum depression mediated by PSE. Using three waves of prospective data, this study constructed an SEM of the interrelationships between feeding patterns, PSE, and postpartum depressive symptoms. Our findings can help in the early prevention and reduction of postpartum depression and may help mothers successfully overcome the high-risk period for postpartum depression.

Our results indicated that Chinese women in this study had severe postpartum depressive symptoms. Approximately one-third of the participants experienced depressive symptoms during the postpartum period, which is consistent with the results reported in other regions of China (Guangzhou, 34.0% [31]; Shenzhen, 27.0% [32]; Shanghai, 23.2% [33]) and is higher than the global average of 14.0% [34]. Although the respondents, sampling methods, and assessment tools of these studies varied, the results of the present study emphasized that postpartum depressive symptoms among Chinese women were a public health issue requiring urgent intervention.

In this study, non-breastfeeding was associated with higher levels of parenting stress and postpartum depressive symptoms. Although breastfeeding is recommended by the World Health Organization and the National Health Commission of China as the best practice for achieving health, its impact on maternal PSE and mental health has not been widely studied [35]. Our research found that breastfeeding not only helped enhance maternal PSE but also protected mothers from the impact of postpartum depressive symptoms, which is consistent with the findings of Botha et al. [36] and Alimi et al. [37]. Clinical experience has shown that breastfeeding promoted hormone secretion, which can protect maternal mental health by reducing a cortisol response to stress [38]. Additionally, skin-to-skin contact during breastfeeding can enhance the mother–infant bond and reduce psychological stress. Breastfeeding also helped to reduce depression, fatigue, and anxiety during parenting. Evidence has shown that breastfeeding mothers report lower levels of sleep disorders, with an average sleep duration of 40–45 min longer than mothers who do not breastfeed [39]. Good sleep patterns and regulatory abilities are also conducive to alleviating parenting stress and preventing postpartum depressive symptoms.

The mediation model established in this study showed that PSE mediated the association between feeding patterns and postpartum depressive symptoms. To some extent, the effect of breastfeeding on preventing postpartum depression was achieved by improving PSE. The reasons may include the following aspects, namely parenting efficacy and satisfaction. First, high levels of PSE enable mothers to perceive sufficient parenting knowledge and skills, thereby enabling them to maintain positive emotional experiences during breastfeeding. PSE was related to mothers' higher sensitivity and lower intrusiveness [40, 41], which meant more physical contact, touch, and intimate interactions during breastfeeding and positive and appropriate responses to infants' needs. Mothers with good role adaptation feel more confident in handling infant growth and development and perceive more enjoyment in breastfeeding. Second, PSE helped breastfeeding mothers perceive more value and satisfaction, thereby preventing anxiety and depression caused by feeding disorders and difficulties in infant care. According to the Stress Coping Theory proposed by Lazarus and Folkman [42], perceived parenting stress stemmed from the threat of stressors (non-breastfeeding) to personal health and well-being, which in turn affected positive emotional states. Breastfeeding helped reduce and alleviate infant temperament and sleep problems [43, 44], enabling breastfeeding mothers to gain a sense of parenting value and achievement. During the postpartum period, improvement in PSE would further strengthen the positive effect of breastfeeding on reducing negative psychological problems, such as irritability and anxiety.

Evidence for the mediating role of PSE in the relationship between breastfeeding and postpartum depressive symptoms in this study provided a new perspective for managing postpartum depression. Previous studies have regarded feeding patterns and PSE as independent predictors of postpartum depression [7, 8, 45, 46]. However, few studies have explored the mechanism of the association among these three variables using a longitudinally tracked mediation model design. The results of this study suggested that providing health education and nursing guidance focused on parenting, improving women's PSE, and reducing their negative self-attributions may be important means for encouraging breastfeeding and preventing postpartum depression. Because both breastfeeding practices and PSE are factors that can be changed and controlled, developing corresponding parenting empowerment plans, such as providing parenting courses and postpartum support related to parenting knowledge and maternal–infant interaction skills, can help further exert the beneficial effects of optimal breastfeeding practices and improve mothers' postpartum mental health. In the future, postpartum health management services based on the Chinese National Essential Public Health Services program should be implemented to create a mutually aid-friendly breastfeeding and parenting environment and social support network, with the aim of decreasing negative emotions such as postpartum depression.

To our knowledge, this was the first study to explain the mediating effect of PSE on the connection between feeding patterns and postpartum depressive symptoms. This study, however, is subject to several limitations. First, this study was conducted only in Hubei Province, China, which somewhat restricts the representativeness of the sample and the generalizability of the findings. Future studies should focus on populations from various regions and different societal backgrounds. Second, maternal feeding behavior, parenting efficacy, and mental health are complex processes that occur after childbirth and are influenced by multiple factors such as childbirth experience, family relationships, and social roles. Therefore, further replication studies with larger sample sizes are warranted. Finally, this study categorized feeding patterns into binary variables: breastfeeding and non-breastfeeding. Mothers may opt for a combination of both, which warrants a trichotomous classification to uncover potential novel insights. Future research should differentiate between exclusive breastfeeding and mixed feeding to further investigate the effects of breastfeeding on maternal mental health.

## 5. Conclusions

PSE mediated the relationship between feeding patterns and postpartum depressive symptoms. The results of this study emphasized the importance of incorporating interventions targeting PSE into mental health strategies, with a particular focus on strengthening the positive effects of breastfeeding. In the future, measures should be developed to improve mothers' adaptability to parenting, reduce stress, and utilize the critical role of breastfeeding practices to prevent postpartum depressive symptoms.

## Figures and Tables

**Table 1:** General characteristics of the 708 women who completed follow-up.

Variables	*N* (mean)	% (SD)
Age	27.14	3.17
Education level		
Junior high school or below	60	8.47
High school	101	14.27
Junior college	505	71.33
Master's degree or higher	42	5.93
Work status		
Employed	427	60.31
Unemployed	281	39.69
Residence		
Urban	593	83.76
Rural	115	16.24
Feeding patterns		
Breastfeeding	364	51.41
Non-breastfeeding	344	48.59

Abbreviation: SD, standard deviation.

**Table 2:** Comparison of postpartum depressive symptoms and PSE based on different feeding patterns.

Variables	Total	Feeding patterns		*t*	*p* Value
		Breastfeeding	Non-breastfeeding		
PPDs, M (SD)	9.72 ± 5.43	9.66 ± 5.31	9.78 ± 5.56	2.419	0.015
Anhedonia	0.91 ± 1.30	0.83 ± 1.23	0.97 ± 1.37	2.318	0.022
Anxiety	4.12 ± 1.94	4.04 ± 1.89	4.20 ± 2.00	2.281	0.025
Depression	4.60 ± 3.03	4.58 ± 3.03	4.62 ± 3.04	1.976	0.048
PSE, M (SD)	70.16 ± 12.34	71.16 ± 12.44	69.10 ± 12.16	2.224	0.026
Efficacy	33.94 ± 6.30	34.46 ± 6.32	33.39 ± 6.23	2.271	0.023
Satisfaction	36.22 ± 7.74	36.70 ± 7.64	35.71 ± 7.82	1.989	0.040

Abbreviations: M, mean; PPDs, postpartum depression symptoms; PSE, parenting self-efficacy; SD, standard deviation.

**Table 3:** SEM fit indices.

Model fit indices	Evaluation criterion	Outcome	Fit situation
RMSEA (95% CI)	<0.08	0.066 (0.055, 0.078)	Good
IFI	>0.09	0.980	Good
CFI	>0.09	0.984	Good
GFI	>0.09	0.967	Good
AGFI	>0.09	0.944	Good
PGFI	>0.05	0.599	Good
PNFI	>0.05	0.501	Good

Abbreviations: AGFI, adjusted goodness-of-fit index; CFI, comparative fit index; CI, confidence interval; GFI, goodness-of-fit index; IFI, incremental fit index; PGFI, proportion of goodness-of-fit index; PNFI, parsimony normed fit index; RMSEA, root mean square error of approximation; SEM, structural equation model.

**Table 4:** The path coefficients of SEM.

Parameter estimates	*β*	95% CI	SE	CR	*p* Value
Structural model					
Feeding patterns → PSE	−0.16	(−0.11, −0.21)	0.01	−14.51	<0.001
PSE → PPDs	−0.60	(−0.56, −0.66)	0.03	−20.83	<0.001
Feeding patterns → PPDs	0.25	(0.18, 0.30)	0.03	7.17	<0.001
Measurement model					
PSE → efficacy	0.65	(0.59, 0.73)	0.08	8.33	<0.001
PSE → satisfaction	0.82	(0.75, 0.88)	0.07	12.05	<0.001
PPDs → anhedonia	0.60	(0.51, 0.67)	0.06	10.83	<0.001
PPDs → anxiety	0.78	(0.71, 0.83)	0.08	9.74	<0.001
PPDs → depression	0.89	(0.83, 0.93)	0.06	15.08	<0.001

*Note*: *β*, estimates of each path coefficient; SE (standard error), a measure of uncertainty in the estimates of the path coefficients. CR (critical ratio) is used to assess the significance of the path coefficients.

Abbreviations: CI, confidence interval; PPDs, postpartum depression symptoms; PSE, parenting self-efficacy.

**Table 5:** Test results of mediation effect.

Pathway	*β*	Bootstrap 95% CI	*p* Value
Feeding patterns → PSE → PPDs	0.10	(0.07, 0.15)	<0.001

Abbreviations: CI, confidence interval; PPDs, postpartum depression symptoms; PSE, parenting self-efficacy.

## Data Availability

The data that support the findings of this study are available from the corresponding author upon reasonable request.
